# Effects of environmental factors and intraspecific niche overlap on the body and ecological characteristics of red-tongued pit vipers (*Gloydius ussuriensis*)

**DOI:** 10.1038/s41598-023-48707-z

**Published:** 2023-12-03

**Authors:** Min Seock Do, Seok-Jun Son, Ji-Hwa Jung, Sang-Cheol Lee, Green Choi, Hyung-Kyu Nam

**Affiliations:** 1 National Institute of Biological Resources, Seo-gu, Incheon, 22689 South Korea; 2 National Science Museum, Daejeon, 34143 Republic of Korea; 3 Honam National Institute of Biological Resources, 99 Gohadoan-gil, Mokpo, 58762 South Korea; 4 Department of Biology, Incheon National University, Incheon, 22012 South Korea; 5 MEET GREEN, Seocheon, 33646 Republic of Korea

**Keywords:** Ecology, Evolutionary ecology, Zoology, Herpetology

## Abstract

The body condition of a snake species provides important physiological, morphological, and ecological information that elucidates its habits, life cycle, and competitive relationships. We measured the body size and condition of the wild *Gloydius ussuriensis* population in South Korea from 2018 to 2022, analyzed the degree of intraspecific niche overlap, and identified the geographic and climatic factors affecting their body condition. We found that the females were longer than the males. The body condition index (BCI) of *G. ussuriensis* differed depending on sex and season; the BCI of the females and males was highest in August and October, respectively. Environmental factors related to altitude and temperature affected the body condition of *G. ussuriensis*; BCI increased as the mean annual temperature and winter temperature increased; however, it increased when the annual temperature range decreased. The mean Pinaka index was 0.96, indicating a high degree of niche overlap; however, the niche overlap among the neonates was less than that among the adults and juveniles. To elucidate the causes of niche overlap and mechanisms behind the intraspecific competition among *G. ussuriensis* individuals, the habitat and utilization of food resources at different development stages of *G. ussuriensis* should be further investigated.

## Introduction

### Importance of research on body condition

The body size of an animal is closely related to its home range^1^, movement speed^2^, body condition^3^, and energy requirement^4^. Information about the body size of an animal is important in animal physiology, morphology, and ecology because it elucidates the habits, life cycle, and competitive relationships of an animal^5–7^. Snakes belong to class Reptilia and are characterized by their unique morphological traits: long body shape and absence of limbs. Instead of using weight, scientists use snout–vent length (SVL), which is less affected by breeding, season, and feeding changes, to measure body size. The slope of weight versus SVL can be used to examine animal habits and compare the body shape of different species^2,8,9^. Thus, information about the weight and length of a snake is important in determining its body condition. Body conditions can be used to identify the breeding and feeding periods of an animal species and determine the health and well-being of each individual^2,3^. Various methods have been proposed for calculating the body condition index (BCI); a method that more accurately reflects the fat and protein stores of an animal was developed by standardizing the body size of an animal regardless of its growth, age, and sex^10–12^. The BCI has been employed in various ecological studies on snakes, including the conservation of endangered species, control of invasive species, and understanding of evolutionary body shapes and behavioral habits^2,3,13^.

### Niches and intraspecific competition among snakes

Snakes occupy a specific ecological niche within their environment for survival and breeding. Information about niches is useful for understanding the biogeographical range and evolution of species^14,15^. Niches can be broadly quantified along the axes of food, space, and time; such a quantification allows the analysis of intra- and interspecific niche overlap and niche separation^16,17^. Snakes have a high ecological and morphological variation not only between species but also between individuals (i.e., males and females; adults and juveniles) ^9,18,19^. Niche overlap applies a strong selective pressure on animals, causing a reduced or unequal allocation of resources due to intraspecific competition, which ultimately leads to regional population decline or extinction due to reduced fecundity^20^. Therefore, research on intraspecific niches plays an important role in conserving ecological niches and understanding competition among the individuals of the same species^1,21,22^.

### Gloydius ussuriensis

*Gloydius ussuriensis* has a broad range spanning Korea, China, and Russia. It has been classified as an endangered species in China and is designated as a protected species (no capture) in Korea to prevent its population from declining due to poaching^23–25^. In Korea, *G. ussuriensis* is widely distributed across the entire inland peninsula as well as in Jeju-do and other islands of various areas. It usually inhabits rivers, valleys, and agricultural land adjacent to mountainous regions at an altitude of 0–1000 m^15,26,27^. It is mainly active from April to October and hibernates from November until March of the succeeding year^25^. Females can store sperm for a long time, are ovoviviparous, and produce 3–10 offspring between August and September; however, they do not produce offspring annually^28^. Numerous studies focusing on the different aspects of *G. ussuriensis*, including studies on morphology^23,28–30^, genetic diversity^31,32^, sexual dimorphism of island populations^33^, food sources^34,35^, toxicity^36^, intraspecific competition^15,17^, and conservation^37^, have been conducted. However, studies focusing on the body condition of and intraspecific competition among *G. ussuriensis* individuals have not yet been conducted.

### Objectives

We conducted this study to identify the general trends in a *G. ussuriensis* in Korea. We measured and analyzed the body shape of *G. ussuriensis*, examine its behavioral habits and life cycle, identify the major environmental factors affecting its body condition, and calculate the intraspecific niche overlap. To attain these objectives, we measured differences in body size depending on age and sex, calculated the BCI of the males and females, and compared the results with the month when the measurements were taken. We also determined the environmental factors—geographic and climatic—in the regions where *G. ussuriensis* is commonly found, analyzed the effects of the major environmental factors on its body condition, and determined the occurrence of intraspecific competition based on the extent of niche overlap by age and sex.

## Results

### Snake capture

A total of 356 *G. ussuriensis* specimens were captured, of which 245 were adults (female: 135, male: 110), 91 were juveniles (female: 38, male: 53), and 20 were neonates (Table 1). There were 32 pregnant females (adult: 31, juvenile: 1; Fig. 1).

**Table 1:** Body mass (M, g), snout–vent length (SVL, cm), and M-to-SVL ratio (M/SVL) of the red-tongued pit viper (*Gloydius ussuriensis*).

Species	N	M ± SE	SVL ± SE	M : SVL ± SE
Adult females	135	56.14 ± 1.67	44.89 ± 0.32	1.24 ± 0.03
Adult males	110	46.46 ± 1.07	44.71 ± 0.32	1.03 ± 0.02
Adults	245	51.79 ± 1.08	44.81 ± 0.23	1.14 ± 0.02
Juvenile females	38	29.50 ± 1.77	35.46 ± 0.61	0.82 ± 0.04
Juvenile males	53	29.64 ± 1.12	36.35 ± 0.42	0.81 ± 0.03
Juveniles	91	29.58 ± 0.99	35.98 ± 0.36	0.81 ± 0.02
Neonates	20	6.63 ± 0.64	20.21 ± 0.55	0.32 ± 0.02

*N* sample size, *SE* standard error.

**Figure 1:**
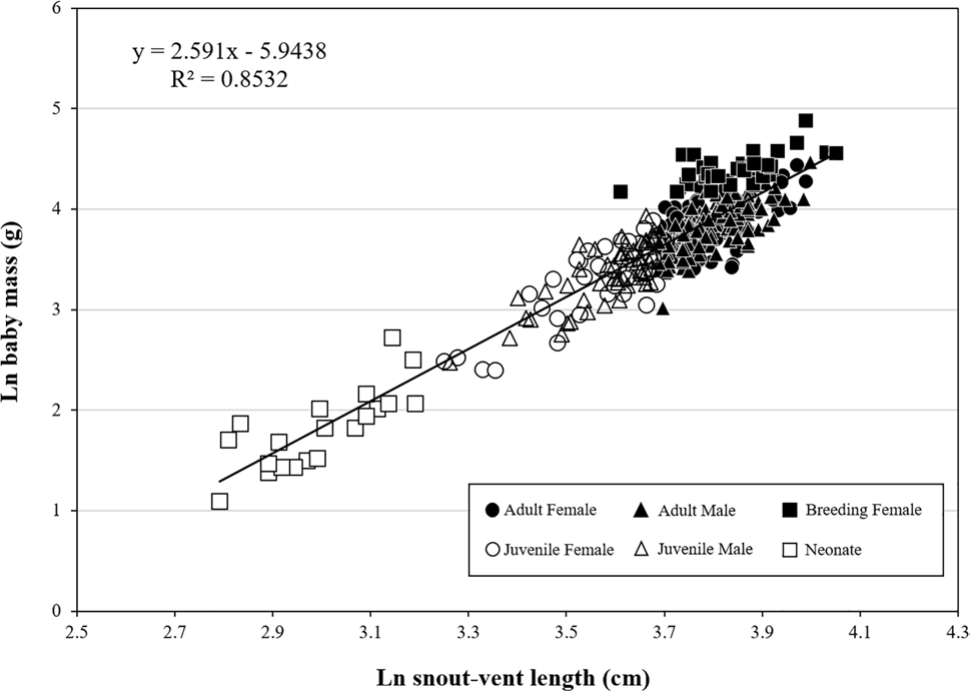
Regression of ln body mass (M; g) on ln snout–vent length (SVL; cm) in red-tongued pit viper (*Gloydius ussuriensis*).

### Body size

The regression equation for ln M on ln SVL was ln M = 2.591 ± 0.057 ln SVL – 5.944 ± 0.211 (r^2^ = 0.853, F_(1,354)_ = 2056.9, *p* < 0.0001, Fig. 1; Table 2). In terms of body size, the adult females (n = 135) had longer SVL than the males [n = 110; 44.89 ± 0.32 cm vs. 44.71 ± 0.32 cm, F_(1,243)_ = 10.94, *p* < 0.0001] and higher mass-to-SVL ratio [1.24 ± 0.03 g/cm vs. 1.03 ± 0.02 g/cm, F_(1,243)_ = 163.30, *p* < 0.0001; Table 3]. The juvenile females (n = 38) did not differ with the males (n = 53) in either SVL [35.46 ± 0.61 cm vs. 36.35 ± 0.43 cm, F_(1,89)_ = 1.495, *p* > 0.05] or mass-to-SVL ratio [0.82 ± 0.04 g/cm vs. 0.81 ± 0.03 g/cm, F_(1,89)_ = 0.05, *p* > 0.05; Table 2].

**Table 2:** Parameters of the linear regressions of ln body mass on ln SVL for red-tongued pit viper (*Gloydius ussuriensis*).

Species	N	*b* ± SE	*a* ± SE	r^2^	*p*-value	SVL_0_	b_RMA_
*G. ussuriensis*	356	2.591 ± 0.057	$-$ 5.944 ± 0.211	0.853	< 0.0001	41.171	2.6950
Viperidae	60	2.655 ± 0.107	$-$ 5.779 ± 0.675	0.91	< 0.0001		2.7833

Included in this table are the average SVL (SVL_0_) and the slope of reduced major axis analysis (*b*_RMA_) used for the calculation of body condition index (see text for explanation). The regression took the form of ln body mass (g) = *b* ln SVL (cm) + *a*. For comparison, the data of the regression equations for Viperidae are also presented (calculated from Feldman and Meiri^38^).

*N* sample size, standard error.

**Table 3:** Results of the principal component analysis of the 19 bioclimatic variables extracted from the regions in South Korea that the red-tongued pit viper (*Gloydius ussuriensis*) inhabits.

Variable		PC1	PC2	PC3
% Variance explained		47.25%	26.97%	17.83%
Eigenvalue		2.996	2.264	1.841
bio1	Annual temperature	**0.291**	0.080	0.241
bio2	Mean diurnal range	**−0.242**	0.233	0.134
bio3	Isothermality	− 0.144	**0.333**	0.110
bio4	Temperature seasonality	− **0.303**	− 0.092	0.128
bio5	Maximum temperature of the warmest period	0.094	0.118	**0.485**
bio6	Minimum temperature of the coldest period	**0.329**	0.004	0.041
bio7	Temperature annual range	− **0.312**	0.040	0.137
bio8	Mean temperature of the wettest quarter	0.168	0.051	**0.454**
bio9	Mean temperature of the driest quarter	**0.320**	0.074	0.058
bio10	Mean temperature of the warmest quarter	0.176	0.037	**0.449**
bio11	Mean temperature of the coldest quarter	**0.319**	0.080	0.094
bio12	Annual precipitation	− 0.018	**0.421**	− 0.032
bio13	Precipitation of the wettest period	− 0.227	**0.282**	0.074
bio14	Precipitation of the driest period	0.114	**0.319**	− 0.228
bio15	Precipitation seasonality	− **0.282**	− 0.119	0.185
bio16	Precipitation of the wettest quarter	− 0.144	**0.370**	0.039
bio17	Precipitation of the driest quarter	0.181	**0.295**	− 0.243
bio18	Precipitation of the warmest quarter	− 0.187	**0.334**	0.076
bio19	Precipitation of the coldest quarter	0.186	**0.286**	− 0.247

The first four principal components (PCs) with eigenvalues larger than one are represented here. The percentages indicate the amount of variation explained by each PC, and the components that were loaded most highly for each parameter are in bold font.

### Body condition

**Figure 2:**
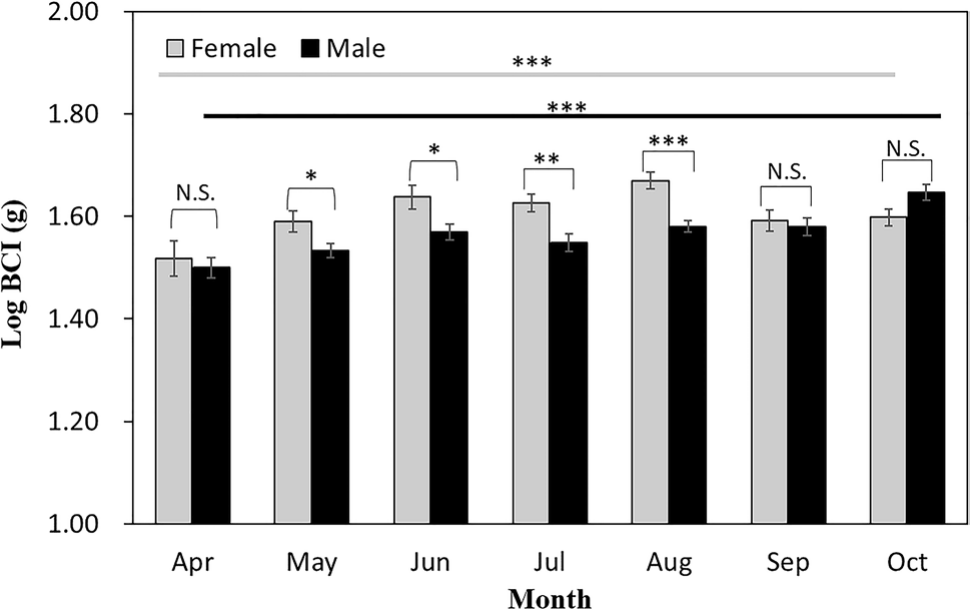
Comparison of body condition of adult female and male red-tongued pit vipers (*Gloydius ussuriensis*) in South Korea according to the month when the measurements were taken using Fisher’s LSD test. *< 0.05, ***< 0.001, and N.S. non significance.

**Figure 3:**
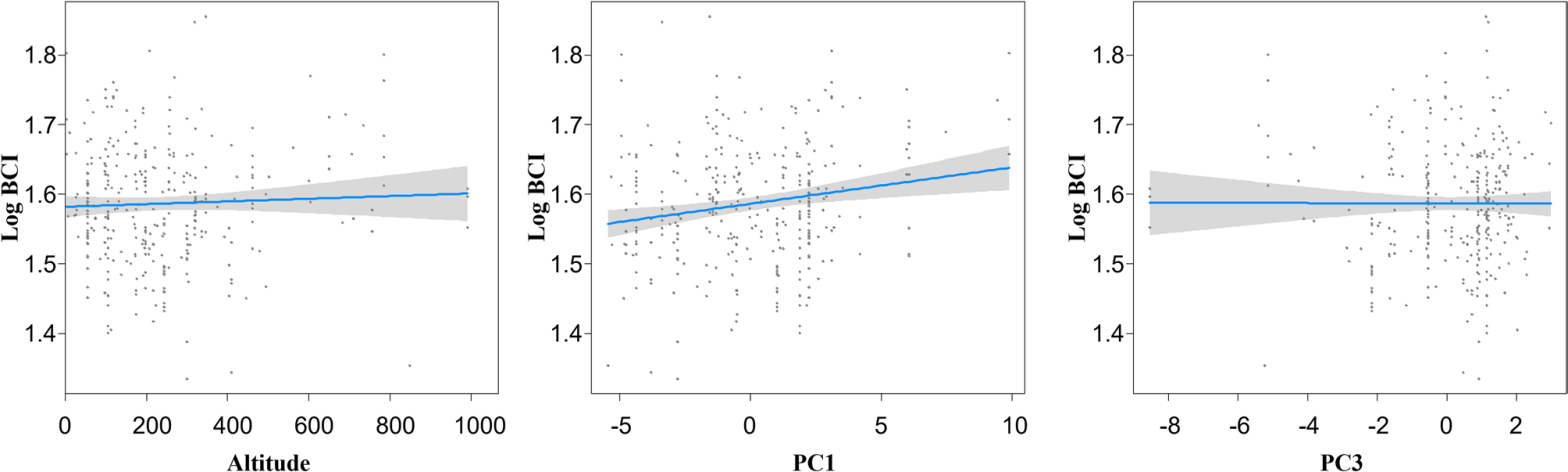
Pattern of association between the body condition of the red-tongued pit viper (*Gloydius ussuriensis*) living in South Korea and altitude, PC1, and PC3.

Body condition had a significant sex [F_(1,334)_ = 27.02, *p* < 0.001] and month [F_(1,334)_ = 6.66, *p* < 0.01], whereas interaction of sex and month had not a significant [F_(1,334)_ = 2.56, *p* = 0.110]. The BCI of the females in August was higher than that in April (*p* < 0.01), May (*p* < 0.01), September (*p* = 0.01), and October (*p* < 0.05; Fig. 3); the BCI of the females in April was lower than that in June (*p* < 0.05) and July (*p* < 0.05; Fig. 3). The BCI of the males in October was higher than that in April (*p* = 0.01), May (*p* < 0.001), June (*p* < 0.01), July (*p* < 0.001), August (*p* < 0.01), and September (*p* < 0.01); the BCI of the males in May was lower than that in August (*p* < 0.05) and September (*p* < 0.05; Fig. 3). The BCI of the females was higher than that of the males in May (*p* < 0.05), June (*p* < 0.05), July (*p* < 0.01), and August (*p* < 0.001; Fig. 2).

### Correlations between climatic factors

When we performed PCA using the climate variables at the locations where *G. ussuriensis* was observed, there were three PCs with eigenvalues > 1, and these explained 92.05% of the variance (Table 3). PC1 best explained the variance in the mean annual temperature, winter temperature, and annual temperature range. When PC1 increased, the mean annual temperature and winter temperature increased; however, the annual temperature range decreased. PC2 was mostly correlated with precipitation-related variables. When PC2 increased, the mean annual precipitation, summer precipitation, and winter precipitation increased. PC3 mostly explained summer temperature, which increased as PC3 increased (Table 3).

### Variables affecting body condition, body mass (g), and SVL (cm)

We found that the environmental factors affecting *G. ussuriensis* body condition were altitude, PC1, and PC3 and body mass and SVL were altitude, PC1, PC2, and PC3 (Table 6). The BCI of *G. ussuriensis* increased as PC1 increased (Fig. 3).

### Intraspecific niche

**Figure 4:**
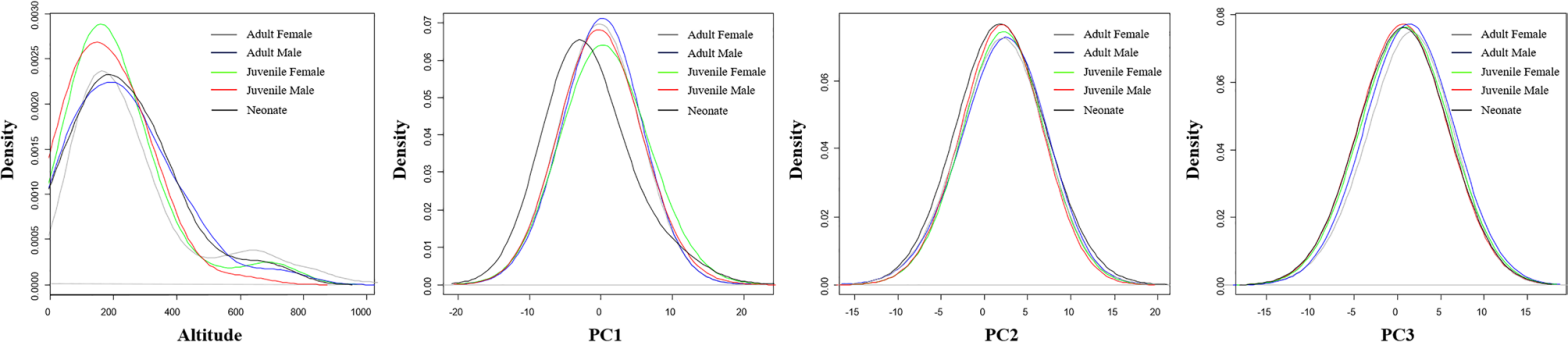
Smoothed frequency distributions (kernel density plot) of the altitude and three principal components (PC1, PC2, and PC3) that the red-tongued pit viper (*Gloydius ussuriensis*) occupies according to its development stage and sex.

The degree of intraspecific niche overlap was extremely high; the probability densities measured for altitude and the three PCs revealed a high degree of overlap (Fig. 4). These results were reflected in the PI, which is a measure of ecological niches based on geographic variables (Table 4). The intraspecific PI was highest for habitat (PI: 0.994; *p* < 0.001), followed by PC3 (PI: 0.973; *p* < 0.001), altitude (PI: 0.971; *p* < 0.001), PC2 (0.957; *p* < 0.001), and PC1 (0.886; *p* < 0.01; Table 6). There was a high degree of overlap between the juvenile females and males for altitude, habitat, PC1, and PC2; in contrast, there was a low degree of overlap between the juveniles (both male and female) and neonates for the abovementioned variables (Table 4). For PC3, the niche overlap between the juvenile females and neonates was the highest, while that between the adult and juvenile males was the lowest (Table 4).

**Table 4:** The Pinaka index (PI) values show the degree of overall niche overlap according to the developmental stage and sex of red-tongued pit viper (*Gloydius ussuriensis*) in South Korea.

	Species	PI	Mean of PI		ES^b^	*p*	Variance of PI		*p*
			Observed	Simulated^a^			Observed	Simulated	
Altitude	Adult female vs adult male	0.9803	0.9712	0.5613	4.1963	< 0.0001	0.0007	0.0971	< 0.001
	**Adult female vs juvenile female**	0.9945							
	**Adult female vs juvenile male**	0.9887							
	Adult female vs neonate	0.9414							
	Adult male vs juvenile female	0.9803							
	Adult male vs juvenile male	0.9773							
	Adult male vs neonate	0.9867							
	**Juvenile female vs juvenile male**	0.9989							
	Juvenile female vs neonate	0.9352							
	Juvenile male vs neonate	0.9299							
Land	Adult female vs adult male	0.9987	0.9944	0.3147	5.3996	< 0.001	0.0001	0.1575	< 0.01
	**Adult female vs juvenile female**	0.9991							
	Adult female vs juvenile male	0.9981							
	Adult female vs neonate	0.9919							
	Adult male vs juvenile female	0.9986							
	**Adult male vs juvenile male**	0.9991							
	Adult male vs neonate	0.9886							
	**Juvenile female vs juvenile male**	0.9989							
	Juvenile female vs neonate	0.9855							
	Juvenile male vs neonate	0.9853							
PC1	**Adult female vs adult male**	0.9949	0.8861	0.4917	4.3072	< 0.01	0.0173	0.0867	< 0.01
	Adult female vs juvenile female	0.9744							
	**Adult female vs juvenile male**	0.9921							
	Adult female vs neonate	0.7727							
	Adult male vs juvenile female	0.9761							
	Adult male vs juvenile male	0.9899							
	Adult male vs neonate	0.7051							
	**Juvenile female vs juvenile male**	0.9937							
	Juvenile female vs neonate	0.7119							
	Juvenile male vs neonate	0.7502							
PC2	Adult female vs adult male	0.9815	0.9567	0.5299	4.9986	< 0.001	0.0022	0.0740	< 0.001
	**Adult female vs juvenile female**	0.9979							
	**Adult female vs juvenile male**	0.9947							
	Adult female vs neonate	0.8897							
	Adult male vs juvenile female	0.9722							
	Adult male vs juvenile male	0.9914							
	Adult male vs neonate	0.9570							
	**Juvenile female vs juvenile male**	0.9936							
	Juvenile female vs neonate	0.8712							
	Juvenile male vs neonate	0.9187							
PC3	Adult female vs adult male	0.9467	0.9727	0.4531	5.2869	< 0.0001	0.0005	0.1001	< 0.0001
	Adult female vs juvenile female	0.9833							
	Adult female vs juvenile male	0.9823							
	**Adult female vs neonate**	0.9894							
	Adult male vs juvenile female	0.9821							
	Adult male vs juvenile male	0.9206							
	Adult male vs neonate	0.9661							
	Juvenile female vs juvenile male	0.9727							
	**Juvenile female vs neonate**	0.9972							
	**Juvenile male vs neonate**	0.9867							

^a^The number of iteration for stimulation is 10,000. ^b^Standardized effect size: (Observed index-Stimulated index)/(Standard deviation of simulated indices).

## Discussion

In this study, we measured and compared the body size and condition of *G. ussuriensis* to describe their life cycle and behavioral habits. We also determined the effects of geographic and climatic factors in the habitat of *G. ussuriensis* on its body condition and analyzed the degree of intraspecific niche overlap. The females were longer than the males; the SSD index was 0.004, suggesting that female snakes evolved to be larger than males to improve their reproductive ability. BCI differed depending on sex and month; the females had the highest BCI in August, which is the reproductive season; meanwhile, the males had the highest BCI in October, which is just before hibernation. The environmental factors affecting the body condition of *G. ussuriensis* were the climatic factors PC1 and PC3 and the geographic factor altitude. When PC1 increased, the body condition of *G. ussuriensis* also increased. The mean PI was 0.96, indicating a very high degree of niche overlap according to age and sex.

### Body size

The slope of body size versus weight for *G. ussuriensis* was 2.59, which is similar to the mean slope (2.67) derived from 60 Viperidae species, was within the 95% confidence interval. Moreover, the slope value of 2.59 is also similar to the interspecific allometric slope of 2.52 derived from 166 Colubridae species^2,38^. Generally, snakes that have thicker bodies are less active in foraging than those with wider bodies and are more likely to adopt a feeding strategy of hiding and waiting for prey^9,39,40^. Viperidae species are specialized for ambush hunting^41,42^. Snake species that utilize various food sources move more actively and ambush or hunt less frequently than those with less diverse food sources^43^. For example, among 23 *Bothrops* species (family Viperidae), the two species with the most diverse food sources (6 taxa; *B. atrox* and *B. jararaca*) seek locations with the most abundant food and forage actively; however, they also carry out ambush hunting occasionally^40,44^. The *G. ussuriensis* population in Korea utilize diverse food sources; their main food sources are amphibians and rodents, followed by fish, mammals, reptiles, and invertebrates. On the other hand, *G. intermedius* only feeds on rodents^26,34,45^. According to previous research that utilized wireless tracking devices to study the *G. ussuriensis* population in Korea, the mean travel distance of *G. ussuriensis* (39.64 ± 22.11 m), which moved for 1 month (September), was longer than that of *G. saxatilis* (21.50 ± 23.40 m), which moved for 2 months (August and September^46,47^). In particular, *G. intermedius* is a stationary species, and the frequency of its ambush and hiding reached 92.69% (active 6 times out of 82 observations^48^). *Gloydius ussuriensis* has a thinner body than other Viperidae species and utilize more diverse food sources; hence, it actively forages for prey.

### Sexual size dimorphism

Although SSD index of our *G. ussuriensis* was 0.004, which is a very low value, the fact that females have evolved to be larger than males is meaningful in evolutionary ecology (Table 4). Typically, female snakes require more intra-abdominal space to allow the growth of eggs and embryos; therefore, they have evolved to be larger than males to improve their reproductive ability^9,20,38^. Viperidae species are an exception because the males are typically larger than the females. Previous studies have reported that there are only 6 out of 29 Viperidae species in which the females are larger than the males; these species have an SSD index ranging from 0.01 to 0.15^2,9^. Although Korean *G. ussuriensis* females are larger than males, the difference between the two sexes is minimal, unlike in other Viperidae species. Meanwhile, different SSD indices were computed for the *G. ussuriensis* populations in islands such as Jeju-do ($-$ 0.03) and Gapa-do (0.02)^33^. Such a difference in SSD indices is attributed to the differential evolution of the island populations due to the ecological and geographic characteristics of islands, where environmental factors (food, habitats, climate, etc.) are typically restricted^28^. For example, the main food sources of *G. ussuriensis*, which are amphibians (inland: 23 species, Jeju: 7 species) and rodents (inland: 19 species, Jeju: 8 species), geographically differ in terms of species diversity. Since these animals are more diverse in inland regions than in Jeju-do, there are also differences in the preferred food sources between the inland and Jeju-do populations of *G. ussuriensis*^26,27,35,49^. In the *G. ussuriensis* population in Jeju-do, the males have evolved to be larger than the females due to intrasex competition, as a means of increasing the frequency of mating and occupying favorable breeding grounds; these species also tend to show a higher rate of male-to-male combat behavior^21,50^. Nevertheless, combat behavior among male *G. ussuriensis* has not yet been documented^28,31,51^. According to previous studies, carpet pythons (*Morelia spilotus*) exhibit differences in combat behavior depending on the subspecies (*M. s. spilotus* vs *M. s. variegatus*^52,53^). Hence, further research should be conducted to determine the unknown combat behaviors, in addition to the typical ones (e.g., head raising, dancing, etc.), of adult *G. ussuriensis* males^9^. In summary, the *G. ussuriensis* females in Korea were larger than the males, and it is rare among Viperidae species to have a positive SSD index.

### Body condition

The BCI of *G. ussuriensis* was lowest in April for both males and females and was highest in August and October for females and males, respectively. The reproductive cycle influences the BCI of *G. ussuriensis* females: they form yolks in May, are close to parturition in August (the month when the highest BCI was recorded), and give birth in September^28,54^. Thus, we observed that the females had higher BCI than males between May and August; however, their BCI decreased rapidly after they had given birth in September. Generally, the body condition of female snakes rapidly decline immediately after giving birth, and 1–4 years are required for them to fully replenish their energy^2,55^. On the other hand, the reproductive cycle has different effects on the body condition of male snakes depending on the species^2,7^. For example, there is no difference in body condition among asp viper (*Vipera aspis*) males during the reproductive season in spite of high testosterone levels and courtship behaviors, while the BCI of pygmy rattlesnake (*Sistrurus miliarius*) males increases as testosterone levels increase^7,56^. The testosterone levels of *G. ussuriensis* males begin to increase in May, peak in August, and rapidly decline at the end of September^54^. The body condition of the *G. ussuriensis* males did not differ from May to August, suggesting that they are unaffected by the reproductive season. Unlike females, which require a considerable amount of energy during pregnancy and parturition, males utilize less energy for reproduction; thus, they are able to maintain a higher body condition^18,56^.

It is hypothesized that the males had the highest BCI in October because they would like to increase their survival rate during hibernation. During the long hibernation period, snakes typically live in a harsh environment where they are unable to forage; such harsh living conditions have negative effects on their body condition and survival^[57.58]^. For example, a study that investigated the weight loss and mortality of 98 snake specimens from three species during hibernation and post-hibernation revealed that the striped whipsnake (*Masticophis taeniatus*) had 39% mortality rate and a 9.4% decrease in body weight, the Great Basin rattlesnake (*Crotalus lutosus*) had 34% mortality rate and a 7.6% decrease in body weight, and the Western yellow-bellied racer (*Coluber constrictor mormon*) had 50% mortality rate and an 11% decrease in body weight^57^. Meanwhile, a 3-year experimental study of 950 northern water snake (*Nerodia sipedon*) neonates revealed that their survival rate during hibernation was only 47%^58^. For this reason, snakes must feed as much as possible before hibernating to increase their body condition and improve their survival rate during hibernation^59^. This explanation supports our finding that the BCI of the males was higher in October, which is the time before hibernation, than in the other months. In summary, changes in the body condition of *G. ussuriensis* appropriately reflect their life cycle, including reproduction, hibernation, and feeding behaviors. The BCI of the females and males was lowest in April, when they had woken from hibernation. The BCI of the females was affected by the reproductive cycle and was highest in August, when parturition is close. In contrast, the males were unaffected by the reproductive cycle, and their BCI was highest in October to increase their survival rate during hibernation.

### Climatic factors affecting body condition

The climatic variables affecting *G. ussuriensis* body condition were PC1 and PC3. High mean annual temperature, high winter temperature, and low annual temperature range, which were represented by PC1, were associated with higher BCI (Table 5; Fig. 3). Generally, an increase in the mean annual temperature results in a more active feeding behavior and larger population size of snakes^60,61^. For example, when the annual mean temperature increased, the percentage of the feeding individuals of Western whip snakes (*Hierophis viridiflavus*) increased, and the activity and size of horseshoe whip snakes (*Hemorrhois hippocrepis*) and Montpellier snakes (*Malpolon monspessulanus*) increased^61,62^. Ultimately, an increase in the mean annual temperature results not only in shorter hibernation but also a longer active period and more opportunities for feeding; thus, snakes grow more rapidly and reproduce more frequently, causing an increase in their population size^60^. In addition, the BCI of our *G. ussuriensis* specimens increased when the temperature increased during hibernation. This finding is consistent with that of a previous study wherein the snakes had higher survival rates when the temperature was higher during hibernation^58^. Therefore, a higher annual temperature range may positively affect the body condition of *G. ussuriensis*. However, when the ambient temperature gets too hot, it may cause negative effects on the body condition of snakes by promoting their metabolism, resulting in excessive energy demands^63,64^. We found that PC3, which reflects high summer temperatures, had an effect on body condition; however, the trend was unclear, and the relationship between PC3 and body condition should be investigated further through long-term monitoring. Meanwhile, the body condition of *G. ussuriensis* was unaffected by PC2, which reflects precipitation-related variables. Such a finding is attributed to the greater influence of altitude and ambient temperature than water sources on maintaining the body condition of *G. ussuriensis*^15,17,26^, although the results of GLMM did not show significance (Table 5). Moreover, researchers who investigated the effects of the surrounding environment on snakes reported that precipitation-related variables had lesser effect on the range, activity, or population size of snakes than other geographic variables^15,61,65^. In summary, temperature was the main climatic variable that affected the body condition of our *G. ussuriensis* specimens; their BCI increased as the mean annual temperature and winter temperature increased.

**Table 5:** Generalized linear mixed model of the effects of geographical and climatic factors on the body condition, weight, and SVL of red-tongued pit viper (*Gloydius ussuriensis*).

Response variable	Explanatory variables	*df*	*χ*^2^	*Pr* (> Chisq)
Body condition	Altitude	1	10.220	***p* < 0.001**
	Land cover	1	1.710	0.191
	PC1	1	19.350	***p* < 0.0001**
	PC2	1	0.025	0.872
	PC3	1	6.919	***p* < 0.01**
Body mass (g)	Altitude	1	30.577	***p* < 0.0001**
	Land cover	1	0.0015	0.9692
	PC1	1	22.844	***p* < 0.0001**
	PC2	1	38.022	***p* < 0.0001**
	PC3	1	13.555	***p* < 0.001**
SVL (cm)	Altitude	1	16.342	***p* < 0.0001**
	Land cover	1	0.2998	0.584
	PC1	1	11.291	***p* < 0.0001**
	PC2	1	26.836	***p* < 0.0001**
	PC3	1	7.6137	***p* < 0.05**

Significant results at *p* < 0.05 are indicated in bold font. The survey year and month used as the random effect.

### Intraspecific niche overlap

Altitude was found to be the geographic variable with significant effects on the health of *G. ussuriensis*. Among the environmental variables, altitude had the greatest effect on the range of three Viperidae species living in Korea, and interspecific ecological niches are delimited by the preferred altitude range of each species^15,17,37^. For example, *G. ussuriensis* mainly inhabits low-altitude mountainous forests below 500 m, *G. blomhoffii* usually inhabits low-altitude rivers and grasslands below 300 m, and *G. saxatilis* typically inhabits high-altitude mountainous forests above 400 m^27,47^. Due to differences in their preferred environments, an interspecific niche separation by altitude (PI: 0.27) and habitat (PI: 0.26) was determined between *G. ussuriensis* and *G. saxatilis*^17^. On the other hand, since the ecological habitats of the *G. ussuriensis* specimens examined in this study were similar among different sexes and ages, there was a very high degree of intraspecific niche overlap by altitude (PI: 0.97) and habitat (PI: 0.99). Nevertheless, the niche overlap, defined by geographic and climatic variables selected by sex and age, between the neonates and adults or between the neonates and juveniles was less than that among adults or between the adults and juveniles. This result is attributed to the observation that the major food sources and habitat types differed by age^5,18,22^. For example, a study on food source utilization by age in Terciopelo pit vipers (*B. asper*) revealed that the neonates prefer amphibians and centipedes, while the adults and juveniles prefer mammals^66^. Meanwhile, turtle-headed sea snakes (*Emydocephalus annulatus*) show differences in habitat with age: the neonates favor shallow areas with ample rubble, while the adults favor deeper areas with more sand and coral^22^. This result is attributed to the effect of snake size on the type and size of utilizable food source and their habitat preference; hence, these differences reduce intraspecific competition^67^. In summary, *G. ussuriensis* had a higher degree of intraspecific niche overlap depending on altitude, habitat, and climatic factors; the niche overlap among the neonates was less that than among the adults and juveniles.

## Conclusion

The analysis of the body size of *G. ussuriensis* specimens revealed that they have thinner bodies than other Viperidae species, which allows them to move actively and hunt diverse food sources. Unlike typical Viperidae species, the SSD index of *G. ussuriensis* was positive, meaning that that females are larger than the males. The difference between the body size of males and females was small. Male-to-male combat behavior, which is related to size, has not yet been observed among *G. ussuriensis* males; thus, further behavioral studies are required. Furthermore, phylogenetic, morphological (e.g., scale count, head length, etc.), and genetic research should be conducted on the Jeju-do population, which had a negative SSD index. Changes in body condition appropriately reflected the life cycle of *G. ussuriensis* in a sex-specific manner, including reproduction, hibernation, and feeding. Among the climatic variables affecting body condition, the annual mean temperature and winter temperature were positively correlated with body condition because *G. ussuriensis* requires an appropriate temperature to maintain its high survival rate while hibernating in harsh environments. Although we excluded pregnant females that were close to parturition from the analysis of the environmental variables affecting body condition, we could not completely exclude the effects of pregnancy on body condition because *G. ussuriensis* females do not reproduce annually, and we could not perfectly detect yolk formation in the early stage of the reproductive cycle in May. There was an extremely high degree of niche overlap between the sexes and development stages of *G. ussuriensis*. The preferred food resources and habitats of the neonates differ from those of the other age groups; hence, the neonates had less niche overlap than the adults and juveniles The habitats and utilization of food resources by *G. ussuriensis* of different development stages should be investigated in more detail so that the causes of niche overlap and the mechanisms involved in intraspecific competition will be revealed.

## Methods

### Survey area

**Figure 5:**
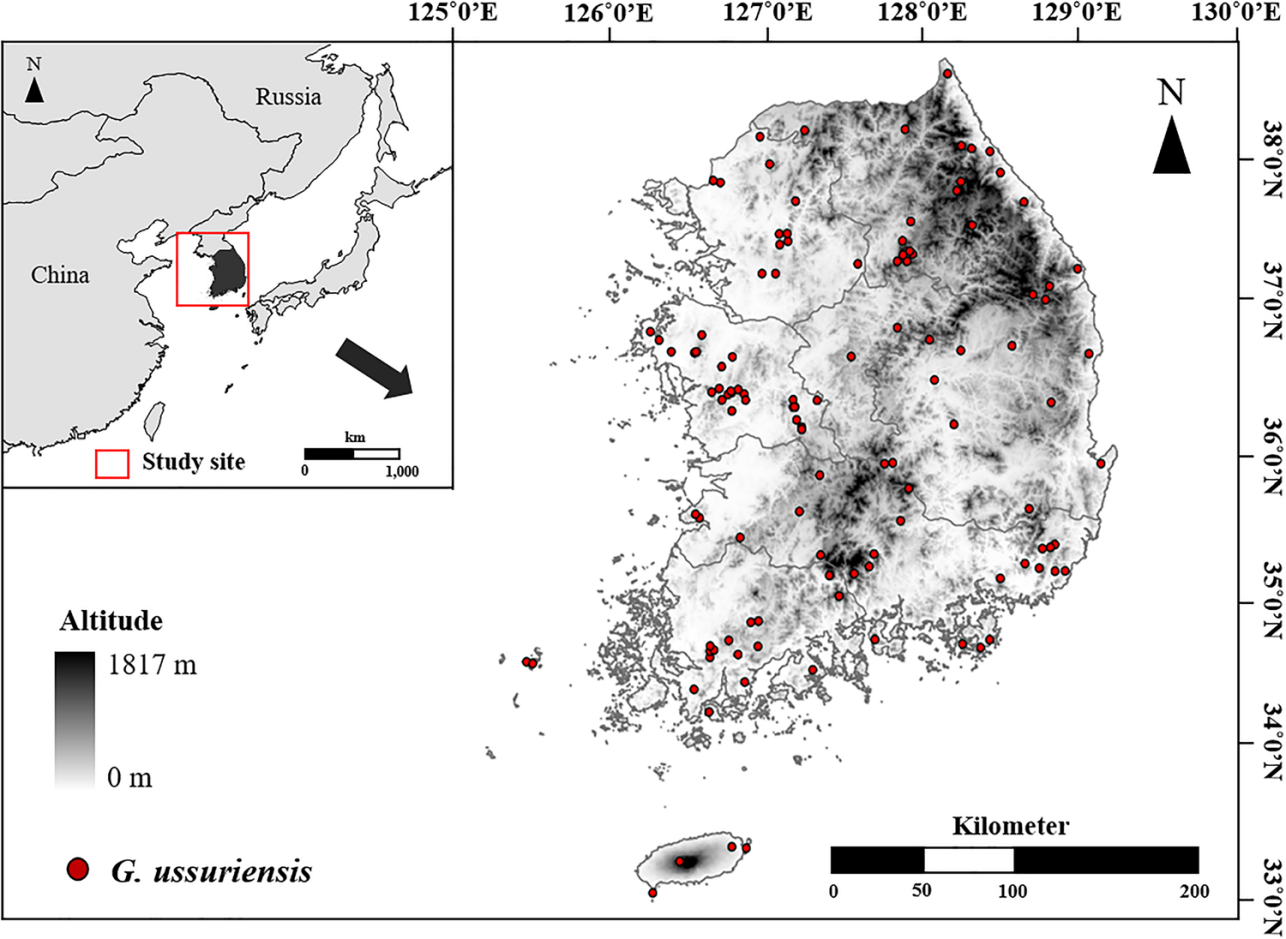
Topographic map of South Korea including its largest island, Jeju.

The survey area was the Korean peninsula. Its altitude ranges from 0 to 1950 m: it is high in the eastern region due to the Taebaek Mountain Range; however, it is low in the western region, where paddies, wetlands, and other flat lands are abundant. The land area consists of 65% forest land and 20% agricultural land^68^. The southern regions include numerous islands of different areas, including Jeju-do (Fig. 5). The mean annual temperature is 13.3 °C, and the annual precipitation is 1244.5 mm. The peninsula has continental climate, with four distinct seasons; it is cold and dry in winter and hot and humid in summer^69^.

### Collection and measurement

The survey was conducted from 2018 to 2022, particularly from April to October. We traveled by foot around valleys, rivers, and agricultural land in the mountainous regions, where *G. ussuriensis* is commonly found^17,26^, across the entire Korean peninsula and randomly selected 123 locations for sampling. We collected snake specimens during the day (09:00–18:00) and at night (20:00–01:00). The snakes were captured by hand and through the use of snake tongs or snake hooks. GPS data were used to record the coordinates and date of capture. The SVL, weight, and sex of the specimens were measured using a flexible ruler (to 0.1 cm), spring or balance (to 0.01 g), and ball-tip probe, respectively^47^. After the measurements, all specimens were released to the same locations where they were caught. To minimize error and improve accuracy, all measurements were taken by three experts who have been studying reptiles for over 20 years (MS, JH, and SC). The capture and measurement of the body size and condition of *G. ussuriensis* were performed after we had received approval from the Institutional Animal Care and Use Committee of the National Institute of Biological Resources (No. NIBR IACUC 20220001). Sample collection, measurement, and analysis were performed in accordance to the guidelines and regulations of NIBR IACUC.

Based on SVL, the development stages of *G. ussuriensis* are classified into neonate, juvenile, and adult^8^. Neonate (< 1 year old), juvenile, and adult specimens are less than 25 cm long, less than 40 cm long, and 40 cm or longer, respectively^54^. The weight of offspring has significant effects on the body condition of pregnant females. We identified the pregnant females by feeling the developing offspring in their abdomen and recognizing the signs of impending parturition (e.g., late gestation: 30–37 days). The female specimens whose reproductive stage (e.g., yolk stage, early gestation: 19–27 days) was uncertain were not included among the pregnant females^6,51,55^.

### Body size and body condition index

To determine the body size of *G. ussuriensis*, the mass-to-SVL ratio was calculated using Eq. (1):

1 $$ln\, M =a\left(\pm SE\right)+b\left(\pm SE\right) ln \, SVL$$

where mass (M, g) is plotted against SVL (cm).

Sexual size dimorphism (SSD) was calculated using Eq. (2)^70^:

2 $$SSD =\left(mean\,  SVL\,  of\,  the \, larger\,  sex\, \div \, mean\,  SVL \, of \, the\,  smaller\,  sex\right) -1$$

Generally, this index is negative when males are larger and positive when females are larger; a zero score indicates symmetry.

Typically, the BCI of snakes is measured from the residuals of the ordinary least squares regression of M on SVL^10,11^. In this study, we calculated the “scaled mass index” (${\widehat{M}}_{i}$) based on the reduced major axis (RMA), which can best predict the changes in fat and protein stores of animals and is less affected by errors^2,12,71^. ${M}_{i}$ is the weight of each individual, while ${SVL}_{i}$ and ${SVL}_{O}$ pertain to the SVL of an individual and the mean SVL, respectively. ${b}_{RMA}$ is the scaling exponent estimated by the RMA regression of M on SVL. BCI allows all individuals to be normalized to the same body size, and is not affected by growth, sex, or age. BCI was calculated using Eq. (3)^71^:

3 $${\widehat{M}}_{i}={M}_{i}{\left[\frac{{SVL}_{O}}{{SVL}_{i}}\right]}^{{b}_{RMA}}$$

The analysis of variance (ANOVA) of the BCI by year and sex in July and August, when *G. ussuriensis* were abundant and the sample size was large, revealed no statistically significant differences (*p* > 0.5). We assumed that the data collected over several years to compare the monthly BCI were collected quantitatively. The logarithm (log) of the BCI was used to present the final results^2,72^. Factorial ANOVA was employed to investigate the body size and condition of *G. ussuriensis*. The SVL and mass-to-SVL ratio were utilized to compare the body sizes of males and females in each development stage (adult, juvenile), and log BCI was applied to compare the body conditions of males and females at different time points (monthly). Fisher’s LSD test was employed to evaluate the differences between the mean values and identify significant variables. Descriptive results are reported as mean ± standard error.

### Geographical environment

By using ArcGIS (v. 10.3; Esri, California, USA), we projected the locations where *G. ussuriensis* was observed onto geographical and climatic environment maps^15,37^. For the altitude map, we utilized a digital elevation model (cell size: 10 m) provided by the National Geographic Information Institute. For the habitat map, we employed the Global Cover (2021 v. 2.0) map (cell size: 10 m) provided by the European Space Agency. For climate data, we used 19 modern (average 1960–1990) climate maps (cell size: 1 km) obtained from Worldclim v. 1.4 (Table 1). Since climate variables are strongly correlated, we performed principal component analysis (PCA) in this study; in accordance with the Kaiser rule, we included three principal components (PCs) with eigenvalues > 1 (Table 4).

### Environmental variables affecting body condition

BCI is often used to investigate the life cycle (reproduction, hibernation, active period, etc.) and other physiological, ecological, and behavioral characteristics of a species by sex, without the need to differentiate pregnant females^2,3,71^. However, because pregnant females have higher BCI than non-pregnant females and males, we excluded pregnant females (n = 32) from the analysis of environmental factors affecting body condition to minimize error. The main environmental factors affecting the body condition of *G. ussuriensis* were analyzed using a generalized linear mixed model (GLMM) with a Poisson distribution. The dependent variable was log BCI, while the independent variables were habitat, altitude, and the three PCs representing climate (Table 6). The survey year and month used as a random effect, and ANOVA was employed to calculate the likelihood ratio test for the fitted model. For the statistically significant geographic and climatic variables, scatter plots were used to visualize the results and examine the relationships of the environmental factors with the body condition of the specimens.

**Table 6:** Average (range: minimum–maximum) values of the geographical and climatic variables in the distribution area of the red-tongued pit viper (*Gloydius ussuriensis*) in South Korea.

Category	Code	Variables (unit)	Type	*G. ussuriensis*
Topographic environment	alt	Altitude (m)	Continuous	248.03 (0.00–990.00)
Habitat environment	land	Land cover	Categorical	Forest (89.81%), Cropland (4.94%), Grassland (4.32%), Shrubland (0.93%)
Climate environment	bio1	Annual mean temperature (℃)	Continuous	11.31 (7.19–15.95)
	bio2	Mean diural range (℃)	Continuous	9.08 (5.46–11.31)
	bio3	Isothermality (%)	Continuous	26.48 (18.62–30.54)
	bio4	Temperature seasonality (SD × 100; ℃)	Continuous	933.68 (709.90–1045.13)
	bio5	Maximum temperature of the warmest month (℃)	Continuous	27.63 (23.80–29.50)
	bio6	Minimum temperature of the coldest period (℃)	Continuous	$-$ 6.48 (-13.00–3.50)
	bio7	Temperature annual range (℃)	Continuous	34.10 (25.70–38.30)
	bio8	Mean temperature of the wettest quarter (℃)	Continuous	22.20 (18.45–24.23)
	bio9	Mean temperature of the driest quarter (℃)	Continuous	$-$ 0.31 ($-$ 5.83 to 9.70)
	bio10	Mean temperature of the warmest quarter (℃)	Continuous	22.45 (19.02–24.93)
	bio11	Mean temperature of the coldest quarter (℃)	Continuous	$-$ 0.53 ($-$ 5.83–7.47)
	bio12	Annual precipitation (mm)	Continuous	1,277.50 (740.00–2000.00)
	bio13	Precipitation of the wettest month (mm)	Continuous	279.78 (174.00–356.00)
	bio14	Precipitation of the driest month (mm)	Continuous	26.05 (14.00–56.00)
	bio15	Precipitation seasonality (%CV; mm)	Continuous	79.94 (53.83–102.61)
	bio16	Precipitation of the wettest quarter (mm)	Continuous	690.75 (403.00–916.00)
	bio17	Precipitation of the driest quarter (mm)	Continuous	96.70 (56.00–212.00)
	bio18	Precipitation of the warmest quarter (mm)	Continuous	667.85 (399.00–886.00)
	bio19	Precipitation of the coldest quarter (mm)	Continuous	97.15 (56.00–217.00)

### Intraspecific niche overlap

To analyze the extent of intraspecific niche overlap for *G. ussuriensis*, the specimens were divided into five categories based on sex and development stage (female adults, female juveniles, male adults, male juveniles, and neonates). The Pinaka index (PI), which reflects niche overlap, was calculated in EcoSim using the geographic and climatic variables in the regions where the specimens were observed^73^. A PI of 1.0 indicates complete niche overlap, while a PI of 0 indicates complete niche separation. The statistical significance of the niche overlap was calculated using a random algorithm in EcoSim^73^. To investigate the extent of niche overlap relative to sex and development stage, which could be explained by altitude and PC1–PC3 from PCA, we generated a kernel probability density plot. All statistical analyses were performed using R 4.1.3^74^.

## Data Availability

The datasets generated during and/or analyzed during the current study are available from the corresponding author on reasonable request.
